# Editors’ Note

**DOI:** 10.5195/ijt.2019.6281

**Published:** 2019-06-12

**Authors:** ELLEN R. COHN, JANA CASON

The *International Journal of Telerehabilitation* (IJT) is a biannual journal dedicated to advancing telerehabilitation by disseminating information about current research and practices. The journal is PubMed indexed, and was recently accepted into Scopus, with indexing to follow.

## ISSUE OVERVIEW

The current issue of the multi-disciplinary *International Journal of Telerehabilitation* (IJT) explores five diverse topics that fall under the unifying umbrella of telerehabilitation: telehealth privacy and security; the usability of an immersive augmented reality based system with haptics for synchronous remote musculoskeletal examination; parent perspectives of occupational therapy delivered through telehealth; the use of telehealth in early intervention in Colorado; and the use of telerehabilitation as an effective platform to help manage Low-Back Pain (LBP). We thank our authors and are honored to publish their work.

## ACKNOWLEDGEMENTS

We are grateful for the generous efforts of the IJT reviewers, many of whom have reviewed prospective IJT articles for over a decade. Sections Editor, William E. Janes, OTD, MSCI, OTR/L has been a very impactful reviewer and support to authors.

Thanks also to the publisher of IJT, the University Library System, University of Pittsburgh. 1 We applaud the expertise and the professionalism of Vanessa Gabler, Electronic Publications Manager, Office of Scholarly Communication and Publishing, for her long-term work with IJT.

IJT is currently sponsored by the Rehabilitation Engineering Research Center on Information and Communication Technology Access at the University of Pittsburgh. The RERC is funded by the National Institute on Disability, Independent Living, and Rehabilitation Research (NIDILRR). Volumes 1–7 were sponsored by the Rehabilitation Engineering Research Center (RERC) on Telerehabilitation at the University of Pittsburgh.

## CALL FOR SUBMISIONS

We cordially invite submissions to the Fall 2019 issue by mid-September 2019. IJT accepts original research, case studies, viewpoints, technology reviews, book reviews, and country reports that detail the status of telerehabilitation.

Sincerely,

Ellen R. Cohn, PhD, CCC-SLP, ASHA-F

IJT Editor

Jana Cason, DHSc, OTR/L, FAOTA

Senior Associate Editor

## Footnotes

1 https://www.library.pitt.edu/e-journals

